# Supplementary material for: BIO-SPEC: An open-source bench-top parallel bioreactor system
Source: HardwareX. 2025 Jul 25;23:e00670. doi: 10.1016/j.ohx.2025.e00670 (PMC12341735; doi:10.1016/j.ohx.2025.e00670)
Supplement: MMC S1 — . [file mmc1.pdf]

Supplementary information

HardwareX

BIO-SPEC: an open-source bench-top parallel bioreactor system.

Laurens Parret[1], Kenneth Simoens[1], Jo De Vrieze[2,3], Ilse Smets[1*]

[1]Department of Chemical Engineering, Bio- & Chemical Reactor Engineering and Safety (CREaS), KU Leuven, Celestijnenlaan 200F box 2424, B-3001 Leuven, Belgium.
[2]Centre for Microbial Ecology and Technology (CMET), Ghent University, Frieda Saeysstraat 1, B-9052 Gent, Belgium.
[3]Centre for Advanced Process Technology for Urban Resource Recovery (CAPTURE), Frieda Saeysstraat 1, B-9052 Gent, Belgium.

*Corresponding author. E-mail: ilse.smets@kuleuven.be

Table S1: Comparison of the BIO-SPEC system features with commercial alternatives.

Feature	BIO-SPEC	Eppendorf DASbox^{TM}	Sartorius Biostat^{TM} B
Agitation	Magnetic stirrer	Overhead drive	Overhead drive
Automation capabilities	Basic: temperature, stirring, feeding, gas supply; programmable in Python; extentions for pH control and gravimetric feed are feasible	Advanced: pH, DO, temperature, agitation, gas flow, cascade control	Advanced: pH, DO, temperature, agitation, gas flow, gravimetric feed, perfusion, cascade control
Cost	±6000 EUR for four reactors (1600 EUR fixed cost, 1100 EUR per reactor)	Depends on quote and configuration, order of magnitude >100 k EUR, >20–50 k EUR secondhand (four-fold system),	Depends on quote and configuration, order of magnitude >100 k EUR, >20–50 k EUR secondhand (two-fold system)
Feeding	ON/OFF control of calibrated peristaltic pumps; extension for gravimetric feed is feasible	2–4 feed lines, programmable rates	Gravimetric feed, up to 4 pumps
Flexibility	Modular, customisable headplate, fully open-source	Flexible but proprietary	Flexible but proprietary
Gas control	Basic: manual or simple ON/OFF control; integration with (Bronkhorst^{TM}) MFCs is feasible	Highly configurable: MFCs and gas mixing	Highly configurable: MFCs and gas mixing, up to 20 L min^{-1}
Off-gas condenser	Liquid-free (Peltier)	Liquid-free (Peltier)	Coolant-based (chiller)
Reactor volume	250-1000 mL	60-250 mL (glass or single-use)	0.35-10 L (glass), 0.6-2 L (single-use)
Scalability	Horizontal scaling via multiple units up to 6 parallel reactors (extension is feasible)	Horizontal scaling via multiple units up to 24 parallel bioreactors	Scalable to 10 L (glass) and 2 L (single-use)
Software	Open-source (Python API)	DASware^{TM} control suite	BioPAT^{TM} control suite
Sterility	Autoclavable	Autoclavable or pre-sterilised single-use vessels	Autoclavable or pre-sterilised single-use vessels
Support	Active open-source community	Vendor support	Vendor support
Temperature control	Water bath	Peltier-based, 10-60 °C	Electrical or water-jacketed, 8-80 °C

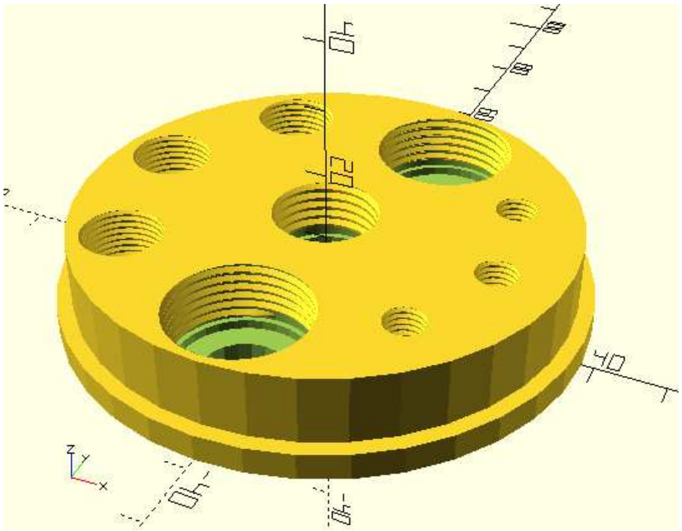

(a) Base design.

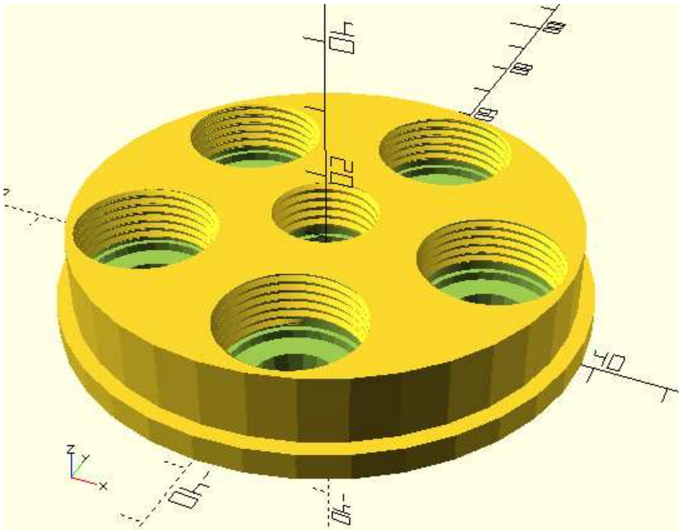

(b) 5-port PG13.5.

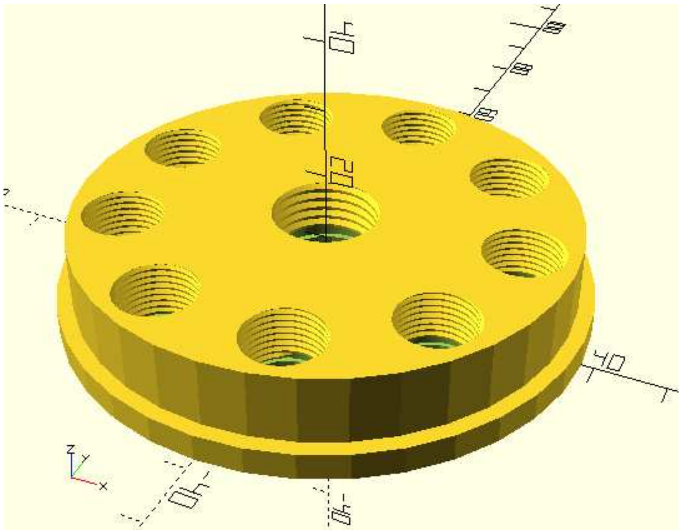

(c) 10-port M12.

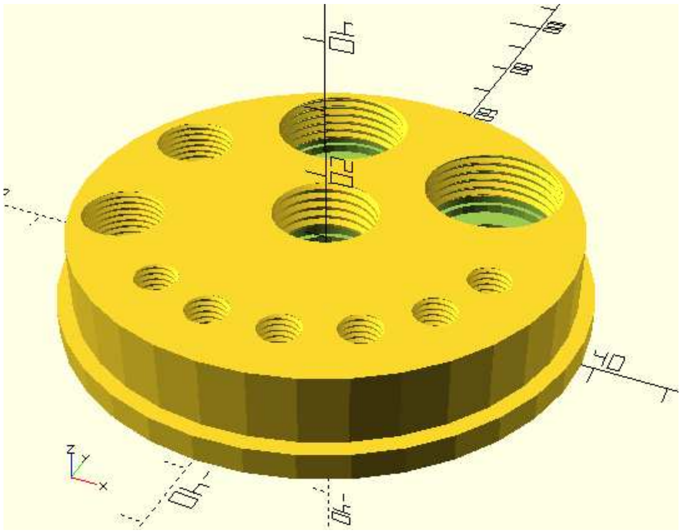

(d) 6-port 1/4-28 UNF, 2-port M12, and 2-port PG13.5.

Figure S1: Examples of the modularity of the headplate design by simply changing the number of ports in the OpenSCAD model. The headplate can accommodate various fittings and configurations, allowing for flexible experimental setups. (a) shows the proposed design in OpenSCAD, while (b-d) show several design variations. By combining these results with the 3D models of the desired fittings, a quick screening test for fitting-headplate compatibility is obtained.

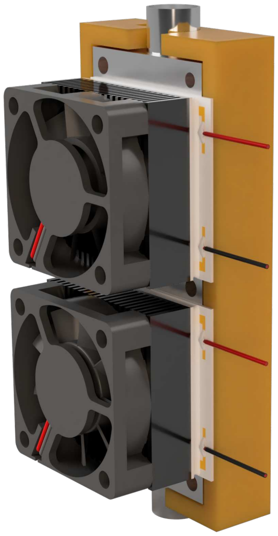

Figure S2: Render of an alternative Peltier condenser design with a smaller footprint. This design is only applicable if the maximum ambient temperature difference is limited.

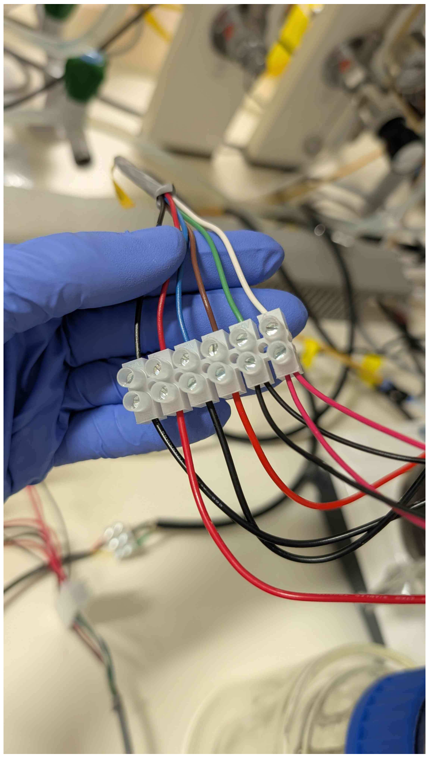

Figure S3: Example of a 6-way terminal strip used to connect the Peltier elements and fans to the power supply.
